# Assessing feasibility, construct validity, and reliability of a new aged care-specific preference-based quality of life instrument: evidence from older Australians in residential aged care

**DOI:** 10.1186/s12955-022-02065-y

**Published:** 2022-12-01

**Authors:** J Khadka, C Hutchinson, R Milte, J Cleland, A Muller, N Bowes, J Ratcliffe

**Affiliations:** 1 Health and Social Care Economics Group, Caring Future Institute, College of Nursing and Health Sciences, Flinders University, Sturt North, GPO Box 2100, Adelaide, South Australia 5001 Australia; 2 Registry of Senior Australians, South Australian Health and Medical Research Institute, Adelaide, South Australia Australia; 3 College of Nursing and Health Sciences, Flinders University, Adelaide, South Australia Australia; 4 Uniting AgeWell, Melbourne, VIC Australia

**Keywords:** Quality of Life, Preference-based measure, Aged care, Residential aged care, Quality of care

## Abstract

**Background:**

Quality of Life-Aged Care Consumers (QOL-ACC) is a new older-person-specific quality of life instrument designed for application in quality assessment and economic evaluation in aged care. The QOL-ACC was designed from its inception with older people receiving aged care services ensuring its strong content validity. Given that the QOL-ACC has already been validated in home care settings and a preference-weighted value set developed, we aimed to assess feasibility, construct validity and reliability of the QOL-ACC in residential aged care settings.

**Methods:**

Individuals living in residential aged care facilities participated in an interviewer-facilitated survey. The survey included the QOL-ACC, QCE-ACC (quality of aged care experience measure) and two other preference-based quality of life instruments (ASCOT and EQ-5D-5L). Feasibility was assessed using missing data and ceiling/floor effects. Construct validity was assessed by exploring the relationship between the QOL-ACC and other instruments (convergent validity) and the QOL-ACC’s ability to discriminate varying levels of self-rated health and quality of life. Internal consistency reliability was assessed using Cronbach’s alpha (α).

**Results:**

Of the 200 residents (mean age, 85 ± 7.7 years) who completed the survey, 60% were female and 69% were born in Australia. One in three participating residents self-rated their health as fair/poor. The QOL-ACC had no missing data but had small floor effects (0.5%) and acceptable ceiling effects (7.5%). It demonstrated moderate correlation with ASCOT (*r* = 0.51, *p* < 0.001) and EQ-5D-5L (*r* = 0.52, *p* < 0.001) and a stronger correlation with the QCE-ACC (*r* = 0.57, *p* < 0.001). Residents with poor self-rated health and quality of life had significantly lower scores on the QOL-ACC. The internal consistency reliability of the QOL-ACC and its dimensions was good (α = 0.70–0.77).

**Conclusions:**

The QOL-ACC demonstrated good feasibility, construct validity and internal consistency reliability to assess aged care-related quality of life. Moderate correlations of the QOL-ACC and other instruments provide evidence of its construct validity and signifies that the QOL-ACC adds non-redundant and non-interchangeable information beyond the existing instruments. A stronger correlation with the QCE-ACC than other instruments may indicate that quality of life is more intimately connected with the care experience than either health- or social-related quality of life in residential aged care settings.

**Supplementary Information:**

The online version contains supplementary material available at 10.1186/s12955-022-02065-y.

## Introduction

Similar to many other developed countries, older Australians with assessed care needs can access government-subsidised and funded aged care services either in their own home or in a residential care facility when they can no longer live independently. In 2020–21, Australian government expenditure on aged care was over AU$23.5 billion, with the largest proportion of funding ($14.3 billion or about 60%) allocated to residential aged care [1]. During this period, over one million older Australians received home-based care and support and just over one third of a million (371,000) accessed residential aged care services [2]. Due to a rapidly increasing ageing population, it is expected that about four million older Australians will need to access care and support from aged care providers either at home or residential aged care by 2050 [3].

A recent federal government commissioned independent investigation by the Royal Commission into Aged Care Quality and Safety in Australia found many systemic failures including substandard service quality, unsafe service delivery, abuse, neglect, and major quality and safety concerns leading to unnecessary distress, suffering, and poor well-being for older people using aged care services [4]. The COVID-19 pandemic has further exposed the cracks in the system with disproportionate levels of COVID-19-related morbidity and mortality in the sector; a situation that is being mirrored internationally, including in Europe, USA and Canada [5–8]. The Royal Commission has made a series of policy recommendations to overhaul the system to achieve transformational changes to ensure dignified, safe, transparent, and evidence-based person-centred care is provided to all older people in aged care. A key recommendation was to mandate routine collection and reporting of quality of life data to complement the existing mandatory clinical quality and safety indicators of care quality (e.g. pressure injury, medication management, unplanned weight loss) [4]. There is a growing consensus that whilst clinical quality indicators are useful, such indicators do not provide individuals’ lived experience of the quality of aged care and its influence on their quality of life [9, 10].

There can be no doubt that the quality of life of older people receiving aged care services is a concern for all Australians. However, to date, quality of life data has not been routinely collected nor reported nationally, and this has created a vacuum in the available evidence base outcomes across the board in terms of tangible improvements to people’s lives to inform policy and practice [11, 12]. In order to introduce person-centred and value-based care reforms across the aged care sector, there is a growing consensus regarding a need to collect data using valid, and reliable, measures of quality of life, where quality of life is encapsulated from the perspective of older people themselves. In order to address this need, our team has developed a new instrument (Quality of Life-Aged Care Consumers, QOL-ACC) for the measurement and valuation of quality of life in aged care [13]. From its inception and at every stage of development, [14–16] we have actively partnered with end users and industry partners. Consequently, the QOL-ACC uniquely incorporates salient quality of life dimensions that are important, meaningful, and preferred by older people receiving aged care services. Importantly, all the quality of life dimensions included in the QOL-ACC are also directly influenceable through the services and supports offered in home and residential care settings by aged care providers [15, 16].

The QOL-ACC is designed to serve as a standalone instrument to encapsulate aged-care specific quality life. It also has the potential to be applied as a preference-based utility measure applicable for assessing cost-effectiveness. In this way, the QOL-ACC can be used to generate evidence for much needed aged care policy reforms to drive efficiency improvements and ensure resources are allocated to maximise quality of life [13].

Although not developed specifically for application in aged care, there are several other existing preference-based instruments that have been widely applied in aged care settings including the EQ-5D-5L and the ASCOT [11, 12]. Our previous work has identified that these existing instruments have a more generic focus on health and social care, and were developed for application with adults of all ages rather than focusing on older people’s perceptions of important quality of life dimensions specific to aged care [11, 12, 17].

The development of a new quality of life instrument from its inception with older people requires a significant investment in time and resources to produce evidence to demonstrate that the instrument is valid, reliable, and produces meaningful scores. As the QOL-ACC is designed to be universally applicable for the aged care sector, assessing its performance in home and residential aged care settings is vital. The QOL-ACC has already been tested for its psychometric performance and construct validity among older people in home care setting [14]. Given its robust developmental origins and successful face validity testing across both home and residential aged care settings, coupled with its strong psychometric performance in home care settings, we hypothesised that the QOL-ACC is a feasible, valid, and reliable instrument to assess aged-care specific quality of life of older people in residential aged care facilities. Therefore, this study aimed to assess the feasibility, construct validity, and internal consistency reliability of the QOL-ACC in a residential aged care population in Australia.

## Methods

### Study sample and the survey

A multi-centre, cross-sectional study was designed and conducted in 22 residential aged care facilities across five Australian states and territories (Australian Capital Territory, Queensland, South Australia, Tasmania and Western Australia). Study participants were aged 65 years and older living in residential care facilities, able to speak and communicate in English, not currently diagnosed with severe cognitive impairment or dementia and determined by the facility manager to have the cognitive capacity to participate. The survey interviews were conducted by trained interviewers experienced in conducting interviews in aged care settings. All of the interviewers in the interview team were trained, skilled and experienced in quality of care experience/quality of life interviews with older aged care residents having recently previously undertaken a large data collection exercise in a similar population of aged care residents for the Royal Commission into Aged Care Quality and Safety investigation [18].

The project manager (CH) provided details of the residential aged care facilities who expressed an interest to participate in the study to the interviewing field teams. The field teams then made contacts with the facilities to arrange a suitable day and time to attend, and also inquired about any COVID-19 related visitor protocols that needed to be observed on each location. On the day of the visit, the facility manager provided the field team with a list of residents who had previously indicated an interest in participation and had given their verbal consent to be approached. Due to the ongoing COVID-19 restrictions in New South Wales and Victoria at the time of the study, interviews were unable to be conducted in these two states (Table 1). All participants provided written informed consent prior to participating in an interview-facilitated survey using a computer assisted personal interviewing (CAPI) device. Family members and next to kin of the residents who expressed their willingness to participate were informed about the research. The interviewers were police checked and trained on ethics and CAPI interview protocols. Each interview was conducted in the resident’s room or in another safe space one-to-one within the residential aged care facility. The study was approved by the Social and Behavioural Research Ethics Committee at Flinders University (Approval no: 5508). The participants completed the QOL-ACC, three other instruments (described below), a series of socio-demographic questions including age, gender, and global items asking participants to self-rate their overall general health (1 item, excellent to poor), and overall quality of life (1 item, excellent to poor) questions. Deidentified data from the CAPI was securely transferred to Flinders University for data storage and analysis.

**Table 1:** Socio-demographic characteristics of the respondents who were living in residential aged care facilities

**Variables**	**Categories**	**Study population (*N* = 200)**	**Australians using residential aged care (*N* = 183, 194)**^a^
**Gender, N (%)**	Female	120 (60.0)	66.3%
	Male	80 (40.0)	33.7%
**Age, N (%)**	65–74	24 (12.1)	10.5%
	75–84	62 (31.2)	29.0%
	85 +	113 (56.8)	59.0%
	Mean Age (SD)	85 (7.7)	
	Median Age (IQR)	86 (80–91)	
	Range	66–100	
**Country of birth, N (%)**	Australia	137 (68.5)	66.0%
	Other	63 (31.5)	33.0%
**Language spoken at home, N (%)**	English	191 (95.5)	91.0%
	Other	9 (4.5)	9.0%
**Facilities states/territories, N (%)**	Australian Capital Territory	8 (4.0)	
	Queensland	31 (15.5)	
	South Australia	92 (46.0)	
	Tasmania	39 (19.5)	
	Western Australia	30 (15.0)	
**Highest educational qualification, N (%)**	No qualifications	69 (34.5)	
	Completed high school	60 (30.0)	
	Undergraduate degree/Professional qualification	35 (17.5)	
	Postgraduate qualification	6 (3.0)	
	Other	30 (15)	
**Self-reported health, N (%)**	Excellent	19 (9.5)	
	Very good	48 (24.0)	
	Good	66 (33.0)	
	Fair	45 (22.5)	
	Poor	22 (11.0)	
**Self-reported quality of life, N (%)**	Excellent	27 (13.5)	
	Very good	52 (26.0)	
	Good	70 (35.0)	
	Fair	37 (18.5)	
	Poor	14 (7.0)	
**SEIFA-IRSEAD quintiles, N (%)**	1 (least advantaged)	31 (15.5)	
	2	12 (6.0)	
	3	34 (17.0)	
	4	45 (22.5)	
	5 (most advantaged)	78 (39.0)	
**SEIFA-IEO quintiles, N (%)**	1 (least advantaged)	43 (21.5)	
	2	0 (0.0)	
	3	33 (16.5)	
	4	29 (14.5)	
	5 (most advantaged)	95 (47.5)	
**QOL-ACC**	Mean ± SD	0.74 ± 0.24	
	Median (IQR)	0.80 (0.65, 0.91)	
	Range	-0.56 to 1.0	
**QCE-ACC**	Mean ± SD	0.91 ± 0.10	
	Median (IQR)	0.94 (0.86, 0.99)	
	Range	0.38 to 1.0	
**ASCOT**	Mean ± SD	0.69 ± 0.13	
	Median (IQR)	0.73 (0.64, 0.79)	
	Range	0.12 to 0.82	
**EQ-5D-5L**	Mean ± SD	0.55 ± 0.37	
	Median (IQR)	0.64 (0.33, 0.52)	
	Range	-0.60 to 1.0	
**EQ-VAS**	Mean ± SD	68.1 ± 21.1	
	Median (IQR)	75 (50,80)	
	Range	10 to 100	

*SEIFA-IRSEAD* Social Economic Indices for Areas- Index of Relative Socio-Economic Advantage and Disadvantage, *SEIFA-IEO* Social Economic Indices for Areas- Index for Education and Occupation, *QOL-ACC* Quality of Life-Aged Care Consumers, *QCE-ACC* Quality of Care-Aged Care Consumers, *ASCOT* The Adult Social Care Outcome Toolkit, *EQ-5D-5L* EuroQuol 5 Dimensions 5 Levels, *EQ-VAS* EuroQuoL Visual Analogue Scale

^a^Australian Institute of Health and Welfare. People using aged care at 30^th^ June 2021. https://www.gen-agedcaredata.gov.au/Topics/People-using-aged-care#Aged%20care%20use%20and%20age. (accessed on 15/06/2022)

### Instruments

#### *QOL-ACC* (Quality of Life-Aged Care Consumers)

The descriptive system for the QOL-ACC was developed using a multi-phase project including extensive work on content development and psychometric testing (including face, content, and construct validity testing) [14–16]. The final descriptive system for the QOL-ACC has 6 dimensions (independence, mobility, pain management, emotional wellbeing, social connection, and activities). Five response levels are attached to each dimension, ranging from the best level ‘all of the time’ to the worst level ‘none of the time’. Responses to the six domains can generate 63,500 possible combinations of response options representing QOL-ACC quality of life states. A discrete choice experiment comprising pairwise comparisons of QOL-ACC quality of life states was undertaken to develop a preference weighted scoring algorithm (value set) based upon the preferences of over 1000 older Australians accessing aged care services [19]. The utility weighted (index scores) for the QOL-ACC ranges from -0.56 (lowest possible score or pit state) to 1.00 (highest possible score). Negative QOL-ACC scores indicate that a small proportion of QOL-ACC states were considered by the majority of older Australians participating in the development of the value set as worse than being dead [19].

#### *ASCOT* (Adult Social Care Outcome Tool)

The ASCOT is a preference based social-care related quality of life instrument [20, 21]. The ASCOT descriptive system has 8 dimensions: personal cleanliness and comfort, food and drink, control over daily life, safety, accommodation cleanliness and comfort, social participation and involvement, occupation, and dignity. Each dimension is framed as “which of the following statements best describes…” and rated on a four response levels representing four different outcome status (‘ideal’, the preferred situation; ‘no needs’, where needs are met; ‘some needs’, where there are needs but no immediate/long-term health implications; and ‘high needs’, where needs have immediate and long-term health implications), [21] and the ASCOT preference weighted scores for English general population range from -0.17 to 1.0, with higher scores representing better social-care related quality of life [20].

#### *QCE-ACC* (Quality of Care-Aged Care Consumers)

The QCE-ACC is a preference-based measure of aged-care-specific quality of care validated in both home and residential aged care settings [18]. The QCE-ACC was developed from a study commissioned by the Royal Commission into Aged Care Quality and Safety in Australia [22]. The QCE-ACC has 6 dimensions (respect and dignity, services and supports, decision-making, staff skills and training, social relationships, and complaints). Each dimension is rated on a 5-response options (‘always ‘ to ‘never’). The QCE-ACC preference weighted scores for older people aged 65 years and above ranges from 0 to 1 with higher scores representing better aged-care specific quality of care experience [18].

#### EQ-5D-5L

The EQ-5D-5L is a generic health-related quality of life instrument with two sections: a descriptive system containing five dimensions of health status (mobility, self-care, usual activities, pain/discomfort, and anxiety/depression) on a 5-level scale of severity (‘no problems’ to ‘extreme problems/unable’) and a visual analogue scale (EQ-VAS). It is one of the most widely used multi-attribute utility instruments and various sets of preference-based utility values have been developed across cross-national general population samples [23]. For this study, we used the Australian preference weights developed by Norman et al. [24]. The EQ-VAS is a vertical visual analogue scale of self-reported health which ranges from 0 (‘worst possible health one can imagine’) to 100 (‘best possible health one can imagine’).

#### Feasibility, validity, and reliability assessments

Feasibility, construct validity, and reliability assessments for the QOL-ACC were guided by the Consensus-based Standards for the Selection of Health Measurement Instruments (COSMIN) guidelines [25, 26]. Feasibility of the QOL-ACC was assessed in terms of missing data and floor/ceiling effects. The floor and ceiling effects occur when the QOL-ACC index score were clustered at the lowest (i.e., -0.57) and highest (i.e., 1.0) possible scores respectively. Low levels of missing data (≤ 5%) and floor/ceiling effect of ≤ 15% indicate good feasibility [27, 28].

Construct validity is the degree to which the scores from health/quality of life instruments represent the construct they purport to measure. This was assessed by testing whether the scores of QOL-ACC were consistent with a series of a priori hypotheses in relation to convergent and known-group validity. Convergent validity is the extent to which the QOL-ACC correlated with other instruments to the degree that was expected, and known group validity is the extent to which an instrument discriminates between groups known to be different. Assessing construct validity is an iterative process whereby a series of hypotheses are tested with an assumption that the instrument validly measures the construct it purports to measure. A total of seven a priori hypotheses were developed to appraise construct validity of the QOL-ACC (Table 2). A significant but medium (coefficient range of > 0.30 to 0.70) correlation between the QOL-ACC and other instruments is indicative of good convergent validity, with related constructs expected to have a stronger correlation than unrelated constructs [29]. For example, we hypothesised that the QOL-ACC would demonstrate a stronger relationship with the QCE-ACC (aged care quality experience) and ASCOT (social care related quality of life) than EQ-5D-5L (health related quality of life). Known group validity was assessed by testing the hypotheses that older people with poor self-reported health and quality of life would have lower scores on the QOL-ACC and vice versa. Construct validity was perceived as adequate if more than 75% of the hypothesised relationships, in terms of the directions and strengths of correlations, were supported by the analysis results [29, 30].

**Table 2:** A priori hypothesized association between the Quality of Life-Aged Care Consumer (QOL-ACC) instrument and other related constructs

Hypothesis No	Expected relationship	Achieved
**Convergent validity**		
1	Given that the QOL-ACC demonstrated moderate correlations with ASCOT, QCE-ACC and EQ-5D-5L in home and community based aged care population, similar correlations were expected in residential aged care population. ^a^	Yes
2	The QCE-ACC measures quality of care experience in residential aged care. People who report better quality of care experience in residential aged care may likely to experience better aged-care specific quality of life. Therefore, a stronger but moderate correlation was expected between the QCE-ACC and the QOL-ACC scores	Yes
3	The ASCOT measure social-care related quality of life which is a similar construct to an aged-care specific quality of life measured by the QOL-ACC. Therefore, a moderate correlation was expected between the ASCOT and QOL-ACC scores	Yes
4	The EQ-5D-5L measures health-specific quality of life. Given that the EQ-5D-5L and QOL-ACC have two similar domains (i.e., mobility, pain), a moderate correlation was expected between the EQ-5D-5L and QOL-ACC scores	Yes
5	The EQ-VAS measures an individual’s perception of their health. Good health does not always reflect a better quality of life. Therefore, a weak correlation was expected between the EQ-VAS and the QOL-ACC scores	Yes
**Known-group validity**		
6	People with poor overall quality of life are likely to perceive poor aged-care specific quality of life. It was also expected that people with different levels of self-reported quality of life would have significantly different scores on the QOL-ACC	Yes
7	Residents with poor health are likely to perceive poor aged-care specific quality of life. It was also expected that people with different levels of self-reported health would have significantly different scores on the QOL-ACC	Yes

*QOL-ACC* Quality of Life-Aged Care Consumers, *QCE-ACC* Quality of Care Experience-Aged Care Consumers, *ASCOT* Adult Social Care Outcome Tool, *EQ-5D-5L* EuroQoL-5 dimensions-5 levels, *EQ-VAS* EuroQoL-Visual Analogue Scale

^a^Khadka J et al. Assessing the construct validity of the Quality-of-Life-Aged Care Consumers (QOL-ACC): an aged care-specific quality-of-life measure. Qual Life Res. 2022;31(9):2849-65 [14]

Reliability of the QOL-ACC was assessed by assessing internal consistency reliability using Cronbach’s alpha and item-scale correlations [31, 32]. Cronbach’s alpha demonstrates the degree to which all dimensions of the QOL-ACC tap into different aspects of the overall underlying construct (i.e., aged care-specific quality of life). Cronbach’s alpha between 0.70 and 0.90 indicate sufficient to good internal consistency reliability [31]. Further evidence of internal consistency was assessed by estimating how highly correlated each dimension was with the overall scale [32]. Ideally, the dimension-scale correlations should be similar, and good or higher item-scale correlations (i.e., ≥ 0.20 is good, and ≥ 0.40 is very good) support the notion that the dimensions were good contributors to the overall scale. Given that the QOL-ACC has six-dimensions but intends to produce a single index score, it was expected that all domains score similarly and correlate highly.

### Statistical analysis

Data were analysed using STATA Version 15.1, Stata Corp LLC, Texas, USA. Descriptive statistics were used to summarise socio-demographics and presented as percentages for categorical variables, with mean (standard deviation) or median (interquartile range) for continuous variables. Socio-Economic Indexes for Areas (SEIFA) provides ranking of geographic areas in Australia by their relative socio-economic advantage and disadvantage based on information from the five-yearly census data collected by the Australian Bureau of Statistics. Two SEIFA indexes: Index of Relative Socio-economic Disadvantage (IRSD) and Socio-Economic Index of Education and Occupation (IEO) were estimated based on postcode of residency [33].

The Kruskal–Wallis Test was applied to assess the difference in the EQ-5D-5L, EQ-VAS, ASCOT, and QCE-ACC scores by QOL-ACC dimension levels (test of monotonicity: to indicate that the scores on other instruments increased by response levels across the QOL-ACC dimensions). For convergent validity (to assess the extent to which the QOL-ACC and other instruments measure related constructs), Spearman’s rank absolute correlation coefficients ( ρ and *p* values) were produced because the distribution of the index scores was not normal, as expected in this context (Additional file 1, Fig. 1). The size of correlation coefficients is interpreted as negligible (0.00 to 0.30), low (> 0.30 to 0.50), moderate (> 0.50 to 0.70) and high (> 0.70 to 0.90). These analyses were complemented by locally weighted scatterplot smoothing (LOWESS) techniques. The LOWESS is a form of non-parametric regression which plots a line of central tendency between two measures on a scatterplot (visually) to demonstrate relationship across all the possible score ranges without making assumption about the actual relationships. LOWESS plots were used to visually assess strength and direction of the relationship between the QOL-ACC and other instruments [34]. For known group validity, the Kruskal–Wallis test was used to test differences between the multiple groups. A post-hoc Dunn’s test was carried out following the Kruskal–Wallis test for multiple pairwise comparison between the groups [35]. A total of 7 hypotheses were tested to assess the construct validity of the QOL-ACC. To adjust for multiple testings, we used the Bonferroni technique to set the significance threshold at *p* ≤ 0.05/21 = 0.002.

**Fig. 1:**
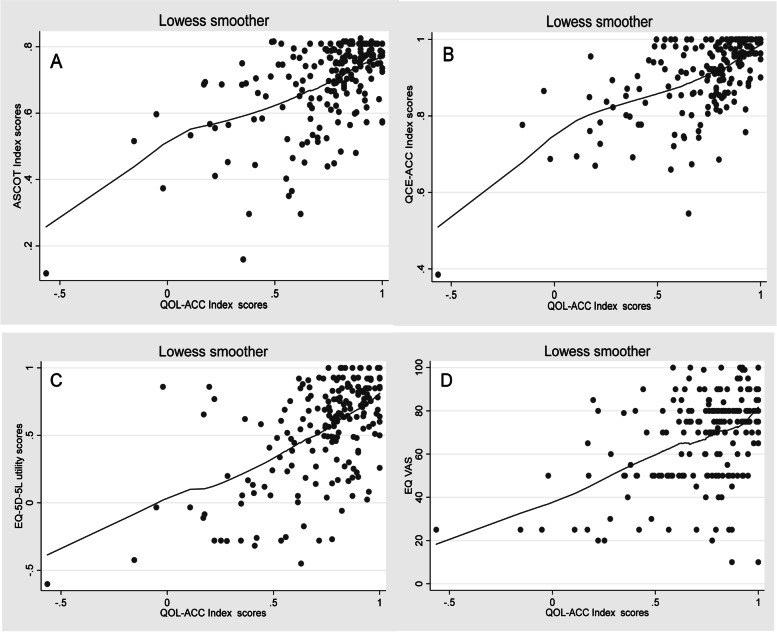
The Quality of Life- Aged Care Consumers (QOL-ACC) LOWESS plots (clockwise) against the Adult Social Care Outcome Tool (ASCOT, **A**), Quality of Care-Aged Care Consumers (QCE-ACC, **B**), EuroQOL 5 dimensions and 5 levels (EQ-5D-5L, **C**) and EuroQOL Visual Analogue Scale (EQ-VAS, **D**)

## Results

Of the 279 residents approached, 55 (19.7%) declined to participate, and six (2.2%) were unable to participate due to sickness. Of the 218 who consented initially, 18 (8.3%) discontinued as they were struggling to comprehend and respond to the survey. Of the 200 respondents who fully completed the survey, 60% were female, mean age was 85 years (SD ± 7.7), 56.8% were 85 years and older, 68.5% were born in Australia and 95.5% had English as a first language. The demographic characteristics of the study population were comparable to the Australian national population in residential aged care as of 30^th^ June 2021 in terms of gender, age, country of birth and language distributions (Table 1) [2]. Based on the one item general health and quality of life questions, a higher proportion of residents self-reported to have fair or poor health (33.5%) compared to fair or poor quality of life (25.5%). Based on the Australian Bureau of Statistics measures of socio-economic conditions, over one in five participating residents were originally from the least advantaged geographic areas (SEIFA IRSEAD quintile 1&2 = 21.5% and SEIFA-IEO quintiles 1 = 21.5%). Participating residents had a higher median index score on the QOL-ACC (median, IQR: 0.80, 0.65–0.91) and the QCE-ACC (median, IQR: 0.94, 0.86–0.99) than on the ASCOT (median, IQR: 0.73, 0.64–0.79) and the EQ-5D-5L (median, IQR: 0.64, 0.33–0.52) (Table 1).

All the respondents completed the QOL-ACC with no missing data, demonstrating its ease of use. The QOL-ACC demonstrated a small floor (0.5%) and acceptable ceiling effects (7.5%). The dimensions of the QOL-ACC mostly demonstrated a monotonic increment in scores on other instruments (ASCOT, QCE-ACC, EQ-5D-5L and EQ-VAS) by their response category levels, signifying that the QOL-ACC response categories were ordered and were able to distinguish between the respondents with low to high scores as measured by other instruments (Table 3).

**Table 3:** Mean and median scores for other instruments by QOL-ACC dimensions and their levels

**QOL-ACC Dimension and Levels (n)**^a^	**ASCOT**		**QCE-ACC**		**EQ-5D-5L**		**EQ-VAS**	
	Mean (SD)	Median (IQR)	Mean (SD)	Median (IQR)	Mean (SD)	Median (IQR)	Mean (SD)	Median (IQR)
**I am able to get around as much as I want to**								
*All of the time* (74)	0.73 (0.10)	0.77 (0.70–0.81)	0.94 (0.09)	0.98 (0.92–1.0)	0.75 (0.23)	0.83 (0.66–0.92)	74.6 (17.5)	75 (65–85)
*Most of the time* (64)	0.69 (0.11)	0.71 (0.63–0.76)	0.90 (0.09)	0.92 (0.85–0.97)	0.62 (0.26)	0.64 (0.49–0.82)	71.2 (18.4)	75 (57–84)
*Some of the time* (28)	0.67 (0.14)	0.71 (0.60–0.79)	0.90 (0.09)	0.91 ( 0.85–0.98)	0.47 (0.34)	0.55 (0.25–0.71)	62.7 (21.9)	70 (50–80)
*A little/none of the time* (34)	0.63 (0.18)	0.68 (0.53–0.76)	0.86 (0.12)	0.89 (0.84–0.94)	0.05 (0.32)	0.05 (-0.26–0.32)	52.2 (23.9)	50 (25–75)
p. *	0.002		< .001		< .001		< .001	
**When I experience pain, it is well managed**								
*All of the time* (110)	0.72 (0.12)	0.77 (0.70–0.81)	0.94 (0.08)	0.97 (0.90–1.0)	0.67 (0.32)	0.78 (0.56–0.91)	72.8 (20.8)	80 (60–90)
*Most of the time* (66)	**0.66 (0.11)**	**0.69 (0.57–0.74)**	**0.88 (0.09)**	**0.89 (0.83–0.95)**	**0.44 (0.36)**	**0.50 (0.23–0.69)**	**63.3 (18.9)**	**70 (50–80)**
*Some of the time* (13)	**0.68 (0.08)**	**0.69 (0.62–0.75)**	**0.89 (0.10)**	**0.92 (0.80–1.0)**	**0.50 (0.29)**	**0.58 (0.48–0.68)**	**66.3 (23.1)**	**70 (50–83)**
*A little/none of the time* (11)	0.57 (0.23)	0.59 (0.55–0.74)	0.84 (0.17)	0.87 (0.78–0.98)	0.10 (0.39)	0.13 (-0.28–0.39)	50.9 (21.4)	50 (25–65)
p. *	< .001		< .001		< .001		< 0.001	
**I am generally happy**								
*All of the time* (59)	0.76 (0.07)	0.78 (9.74–0.81)	0.97 (0.05)	1 (0.96–1.0)	071 (0.28)	0.75 (0.63–0.92)	75.8 (20.5)	80 (65–90)
*Most of the time* (99)	0.69 (0.10)	71 (0.65–0.79)	0.91 (0.08)	0.92 (0.86–0.97)	0.50 (0.37)	0.59 (0.23–0.82)	67.9 (19.4)	75 (50–80)
*Some of the time* (31)	0.64 (0.14)	0.67 (0.53–0.79)	0.86 (0.10)	0.86 (0.80–0.96)	0.47 (0.36)	0.56 (0.20–0.78)	59.0 (20.9)	60 (50–70)
*A little/none of the time* (11)	0.46 (0.20)	0.46 (0.36–0.58)	0.75 (0.15)	0.75 (0.69–0.83)	0.35 (0.52)	0.52 (-0.01–0.77)	52.7 (23.5)	50 (25–80)
p. *	< .001		< .001		< .001		< .001	
**I have as much independence as I want**								
*All of the time* (69)	0.74 (0.10)	0.77 (0.73–0.81)	0.97 (0.05)	0.99 (0.96–1.0)	0.70 (0.26)	0.75 (0.64–0.86)	75.2 (18.1)	80 (70–90)
*Most of the time* (93)	0.69 (0.11)	0.73 (9.65–0.78)	0.90 (0.08)	0.92 (0.85–0.97)	0.55 (0.33)	0.60 (0.37–0.83)	66.3 (20.3)	70 (50–80)
*Some of the time* (18)	0.65 (0.10)	0.69 (0.57–0.72)	0.84 (0.1)	0.85 (0.75–0.90)	0.48 (0.36)	0.58 (0.20–0.77)	71.2 (20.2)	75 (60–85)
*A little/none of the time* (20)	0.55 (0.17)	0.59 (0.43–0.69)	0.81 (0.14)	0.82 (0.77–0.88)	0.10 (0.47)	-0.34 (-0.28–0.62)	48.5 (22.9)	50 (25–67)
p. *	< .001		< .001		< .001		< .001	
**I have good social relationships with family and friends**								
*All of the time* (109)	0.74 (0.09)	0.77 (0.70–0.81)	0.94 (0.06)	0.96 (0.91–1.0)	0.58 (0.34)	0.68 (0.37–0.86)	71.4 (19.7)	75 (60–85)
*Most of the time* (62)	0.68 (0.10)	0.70 (0.61–0.77)	0.90 (0.08)	0.90 (0.85–0.98)	0.57 (0.32)	0.64 (0.48–0.79)	66.6 (21.5)	70 (50–80)
*Some of the time* (21)	0.57 (0.19)	0.62 (0.51–0.68)	0.81 (0.16)	0.84 0.75–0.94)	**0.29 (0.47)**	**0.39 (-0.03–0.62)**	**56.1 (24.8)**	**55 (30–79)**
*A little/none of the time* (8)	0.50 (0.14)	0.45 (0.39–0.63)	0.79 (0.12)	0.75 (0.70–0.88)	**0.57 (0.38)**	**0.76 (0.26–0.86)**	**63.7 (15.3)**	**60 (50–77)**
p. *	< 0.001		< 0.001		0.03		0.04	
**I have leisure activities/hobbies I enjoy**								
*All of the time* (69)	0.73 (0.11)	0.76 (0.70–0.81)	0.95 (0.07)	0.98(0.91–1.0)	0.64 (0.28)	0.69 (0.48–0.86)	72.0 (21.0)	75 (60–90)
*Most of the time* (69)	0.72 (0.10)	0.75 (0.67–0.79)	0.92 (0.07)	0.93 (0.87–0.98)	0.61 (0.31)	0.65 (0.49–0.85)	71.0 (18.7)	75 (60–85)
*Some of the time* (44)	0.65 (0.13)	0.69 (0.58–0.75)	0.88 (0.10)	0.91 (0.79–0.96)	0.43 (0.40)	0.54 (0.14–0.74)	63.4 (20.4)	70 (50–80)
*A little/none of the time* (18)	0.54 (0.18)	0.56 (0.41–0.69)	0.81 (0.15)	0.82 (0.73–0.90)	0.25 (0.54)	0.25 (-0.28–0.77)	53.0 (24.9)	50 (25–80)
p. *	< 0.001		< 0.001		0.006		0.008	

Abnormal values are in bold

^a^The lowest two levels (‘A little of the time’ and ‘None of the time’) were collapsed for analysis due to low cell counts

^*^Kruskal Wallis Test

### Construct validity

#### Convergent validity

The QOL-ACC and the scores from other instruments (QCE-ACC, ASCOT and EQ-5D-5L) were moderately correlated (Table 2 Hypothesis 1 & Table 4). Although it was moderate, the QOL-ACC demonstrated stronger correlation with the QCE-ACC (ρ = 0.57, *p* < 0.001) than with other instruments (Table 2 Hypothesis 2 & Table 4). The QOL-ACC demonstrated similar moderate correlations with the ASCOT (ρ = 0.51, *p* < 0.001; Table 2—Hypothesis 3) and the EQ-5D-5L (ρ = 0.52, *p* < 0.001; Table 2 Hypothesis 4). As expected, the QOL-ACC demonstrated a weaker correlation with the EQ-VAS (ρ = 0.36, *p* < 0.001; Table 2 Hypothesis 5).

**Table 4:** Relationship between Quality of Life-Aged Care Consumers (QOL-ACC) and other instruments

	**Spearman’s correlation co-efficient (*P* values)**	
	**Residential aged care sample (*n* = 200)**	**Home care sample (*n* = 313)**^b^
**ASCOT**	0.51 (< 0.001)	0.61 (< 0.001)
**QCE-ACC**^a^	0.57 (< 0.001)	0.51 (< 0.001)
**EQ-5D-5L**	0.52 (< 0.001)	0.56 (< 0.001)
**EQ VAS**	0.36 (< 0.001)	0.48 (< 0.001)

*QOL-ACC* Quality of Life-Aged Care Consumers, *QCE-ACC* Quality of Care-Aged Care Consumers, *ASCOT* Adult Social Care Outcomes Tool, *EQ5D-5L* EuroQuol 5 Dimensions 5 Levels

^a^QCE-ACC: Australian General Adult Population algorithm used (> 18 years)

^b^Khadka J et al. Assessing the construct validity of the Quality-of-Life-Aged Care Consumers (QOL-ACC): an aged care-specific quality-of-life measure. Qual Life Res. 2022;31(9):2849-65 [14]

LOWLESS graphs (Fig. 1: A, B, C, and D) added further evidence of convergent validity of the QOL-ACC by demonstrating upward trends in the relationship between the QOL-ACC and other instruments, indicating that higher quality of life measured by the QOL-ACC was associated with higher (better) scores on other instruments [34]. The trend was more pronounced between the QOL-ACC and QCE-ACC/ASCOT/EQ-5D-5L (Fig. 1: A, B, and C) than the EQ-VAS (Fig. 1D) across the full range of the QOL-ACC index scores, again mirroring the strength of correlations shown in Table 4.

#### Known group validity

**Fig. 2:**
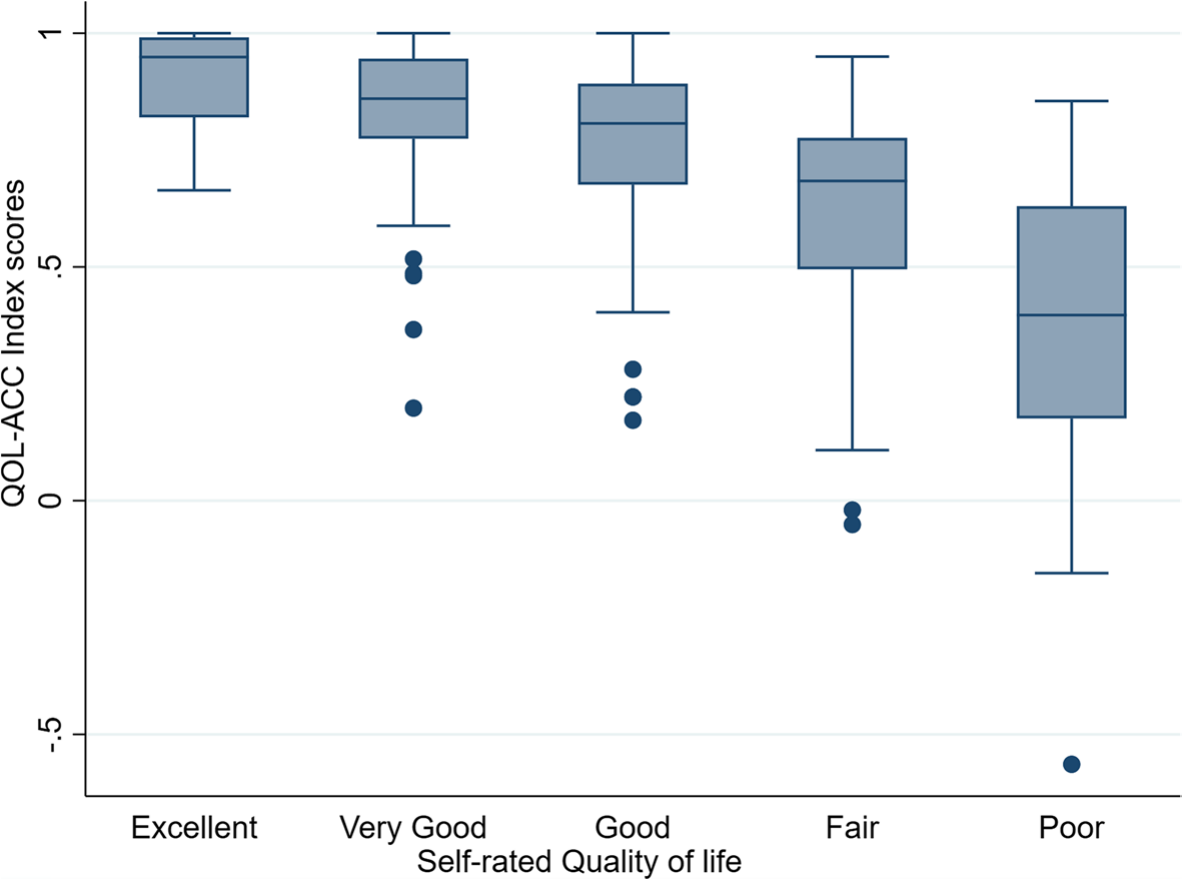
Quality of Life-Aged Care Consumers (QOL-ACC) index scores by self-rated quality of life

**Fig. 3:**
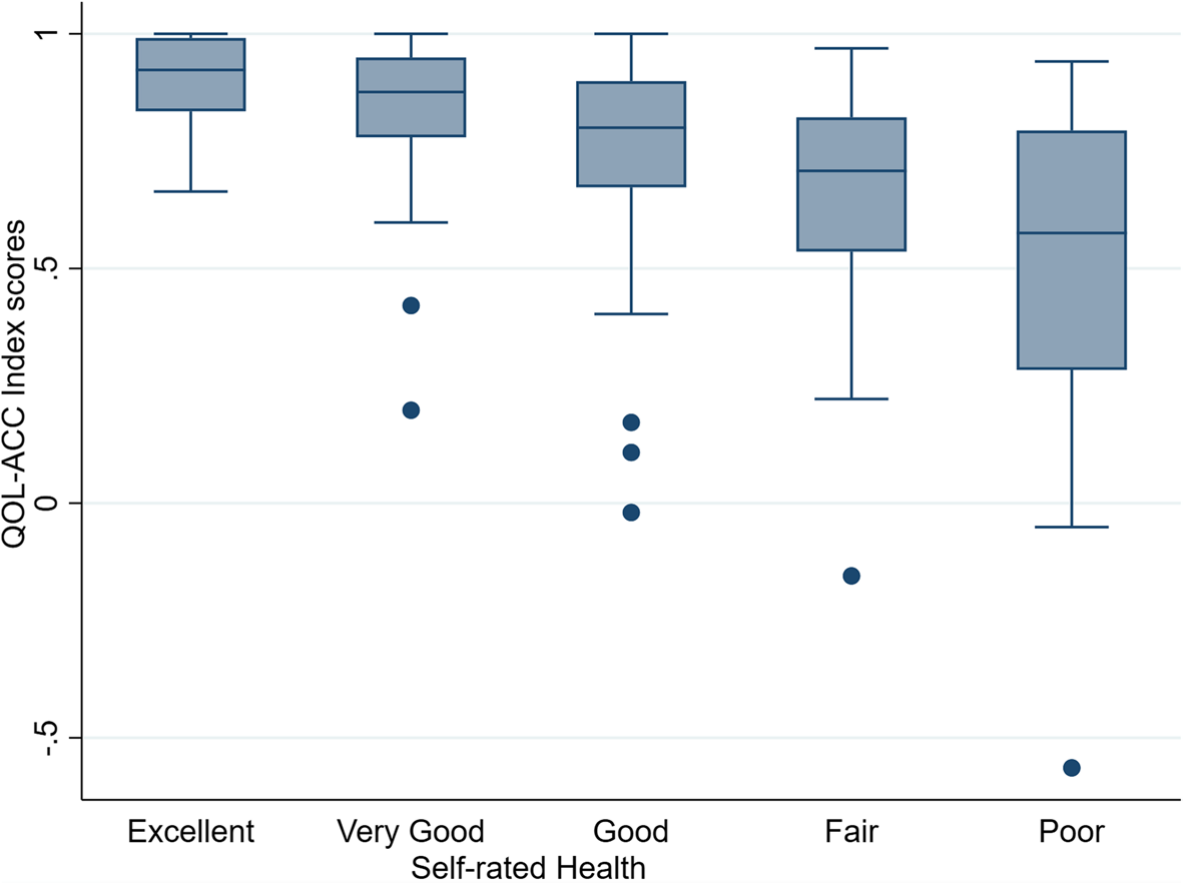
Quality of Life-Aged Care Consumers (QOL-ACC) index scores by self-reported health

Respondents with different ratings of health and quality of life had different scores on the QOL-ACC (Additional file 1, Table 1). More specifically, respondents with poor self-reported QOL had poor index scores on the QOL-ACC (Table 2 Hypothesis 6) with significant differences between all 5 categories of quality of life rating (Fig. 2; *X*^*2*^ = 64.4, *p* < 0.001). Similarly, respondents who reported poor health had poor index scores on the QOL-ACC (*X*^*2*^ = 69.1, df = 3, *p* < 0.001) with significant differences between all 5 categories of health ratings (Fig. 3, *X*^*2*^ = 69.1, df = 3, *p* < 0.001).

All seven a priori hypotheses were confirmed (Table 1), signifying a strong evidence of construct validity of the QOL-ACC in residential aged care population.

#### Reliability

The internal consistency reliability of the overall QOL-ACC (alpha = 0.76) was good. All the QOL-ACC dimensions demonstrated adequate Cronbach’s alpha values with pain management (alpha = 0.77) and independence (alpha = 0.70) dimensions demonstrating the highest and lowest internal consistency respectively (Table 5). Dimension-scale correlations for all dimensions were excellent (≥ 0.40) adding further evidence of internal consistency reliability (Table 5).

**Table 5:** Overall and dimension level Cronbach’s alpha and Pearson’s correlations between individual items (dimensions) and the overall scale (Quality of Life-Aged Care Consumers, QOL-ACC)

QOL-ACC dimensions	Cronbach’s alpha	Dimension-scale correlation
Mobility	0.74	0.62
Pain	0.76	0.58
Emotional well-being	0.71	0.72
Independence	0.70	0.75
Social connection	0.74	0.65
Activity	0.71	0.72
Overall scale	0.77	

## Discussion

This study provides comprehensive evidence of feasibility, construct validity and reliability of the QOL-ACC to assess quality of life among older people living in residential aged care facilities in Australia. These findings are not surprising given how meticulously the content of the QOL-ACC was developed, guided by direct feedback from older people receiving aged care services and industry partners to ensure adequate face and content validity of the QOL-ACC [11, 15, 16]. These findings add further evidence to our previous study in Australian home care settings, in which we demonstrated that the QOL-ACC was a valid instrument to measure quality of life in older people receiving aged care services at home [14]. Therefore, this study provides additional evidence to confirm the validity of QOL-ACC in measuring and valuing quality of life for older people receiving aged care services in Australian home and residential care settings.

Our analyses showed that the QOL-ACC has expected correlations with related constructs. As hypothesised (Table 2) the overall utility scores of the QOL-ACC positively and significantly correlated with the EQ-5D-5L and ASCOT, indicating that aged-care specific quality of life is a similar construct to health-related and social-care specific quality of life respectively. However, the strength of the correlations was moderate, indicating that the QOL-ACC adds new information and is measuring a construct sufficiently different from the constructs measured by existing instruments in aged care settings.

The relationship between the QOL-ACC and other instruments (ASCOT and EQ-5D-5L) in this study is consistent with findings in a parallel matched-design study in older Australian receiving aged care at home [14]. Albeit moderate, the strength of correlation of the QOL-ACC with both the ASCOT and the EQ-5D-5L was higher in the home care population than in this study [14]. These findings may indicate that quality of life among older people in long-term residential aged care is a more complex construct and is influenced by factors that are different than older people living in the community. Simply, when compared to people receiving aged care services at home, aged care residents are likely to be older, more impaired, frail, have multiple morbidities, be highly dependent and have less connection with their peers/loved ones than they would like to have, all of these factors may lead to differing perspectives on quality of life [2, 9, 36].

Older people living in residential aged care facilities are likely to have differing conceptions of quality of life based on their health status, care needs, experience of quality of care, and preferences. Supporting this argument, we found that the QOL-ACC demonstrated the strongest correlation with the QCE-ACC (a measure of quality-of-care experience in aged care) than with EQ-5D-5L and ASCOT (Table 4). These findings contrast with those from our prior work in older people receiving aged care services at home where we found that the QOL-ACC had a weaker correlation with the QCE-ACC than with ASCOT and EQ-5D-5L [14]. Older people in residential aged care facilities are more dependent on daily care supports provided by facilities than older people in the community and may not be able to maintain or improve their quality of life without those services and supports. Therefore, it is possible that quality of life perceptions for aged care residents may be more closely associated with care quality and the care experience than older people living in their own homes. However, the relationship between care experience and quality of life needs further investigation, particularly due to heterogeneity in care needs, as service provision becomes increasingly concentrated on individuals with the greatest care needs. A similar association between care experience and quality of life was reported in a study conducted in the UK [37]. However, it is also important to note that the level of association between the QOL-ACC and QCE-ACC was moderate. This finding supports the argument that these two constructs are distinctive and not directly interchangeable, reinforcing the value of measuring these two different concepts separately [37].

Known group analysis confirmed our prior hypotheses (Table 2) that the QOL-ACC scores were higher in people with higher on global self-reported quality of life (Fig. 2) and health (Fig. 3) items. These findings were mirrored in our previous study that assessed known group validity of the QOL-ACC in older Australians accessing aged care at home [18]. Strong evidence of convergent and known-groups validity demonstrates the construct validity of the QOL-ACC in older people accessing residential aged care services in Australia. Missing data, and ceiling and floor effects, for the QOL-ACC were within acceptable range, and are similar to other studies that have assessed feasibility of other preference-based instruments [28, 38, 39]. Internal consistency reliability for the QOL-ACC was good and the value was similar to literature reports for most of the other instruments, [40, 41] confirming that basic level of reliability of the instrument in residential aged care population. Further ongoing studies will establish other important psychometric properties of the QOL-ACC such as test–retest reliability and responsiveness.

Although this study was carried out in a relatively small sample of 200 older people living in residential aged care services, our study population was broadly representative in terms of age, gender, country of birth, and language distribution when compared with the national population using residential aged care services in Australia (Table 1) [2]. Despite ongoing COVID-19 restrictions at the time of the study, data was collected from five out of eight states and territories in Australia (data was not collected from New South Wales, Victoria and Northern Territory). As the QOL-ACC was primarily designed as a self-reported instrument, we were unable to include older people who had severe cognitive impairment. Although a proxy version of the QOL-ACC is available, due to COVID-19 restrictions operating at the time of data collection, we were unable to extend data collection activities to include proxies for those with severe cognitive impairment.

Our study has provided evidence to support the feasibility, reliability and construct validity of the QOL-ACC in residential aged care settings. Given that psychometric validation of any instrument is a journey than a destination, our future work will explore evidence of its content relevance, reliability (test–retest), validity, responsiveness (i.e., sensitive to detect change) and the utility of the QOL-ACC in a broader aged care population using a novel clinimetics approach. In essence, clinimetrics provides a more suitable conceptual and methodological framework by combining judgements from the stakeholders (e.g. aged care providers and end users) and modern psychometric methods (such as Rasch analysis and/or Item response theory) to assess important psychometric properties such as reliability, responsiveness, clinical validity and utility of the instruments [42, 43].

## Conclusion

The QOL-ACC demonstrated good feasibility, construct validity, and internal consistency reliability to assess aged-care specific quality of life among aged care residents in Australia. With an increasing awareness of the central importance of person-centred outcomes for monitoring quality and safety in aged care, QOL-ACC has the potential to be applied as a key aged care quality indicator. The preference weighted scoring algorithm accompanying the QOL-ACC facilitates its application in economic evaluation for measuring and valuing quality of life to generate new evidence to guide value-based aged care policy and practice and facilitate sector-wide improvements.

## Supplementary Information

**Additional file 1:** **Table 1.** Distribution of the Quality of Life- Aged Care Consumers (QOL-ACC) index scores by self-reported global health and quality of life items. **Figure 1.** Histograms showing distribution of scores of Quality of Life-Aged Care Consumers (QOL-ACC, 1A), Adult Social Care Outcome Tools (ASCOT, 1B), Quality of Care-Aged Care Consumers (QCE-ACC, 1C), EQ VAS (1D) and EQ-5D-5L (1E).

## Data Availability

No (under embargo).

## References

[1] Australian Institute of Health and Welfare. Canberra: Spending on aged care; 2022. (https://www.gen-agedcaredata.gov.au/Topics/Spending-on-aged-care).

[2] Australian Institute of Health and Welfare. People using aged care: at 30 June 2021 (https://www.gen-agedcaredata.gov.au/Topics/People-using-aged-care). Canberra. 2022

[3] Parliament of Australia. Reforming Australia's aged care system: are we there yet? (https://www.aph.gov.au/About_Parliament/Parliamentary_Departments/Parliamentary_Library/pubs/BriefingBook44p/AgedCare). Canberra. 2013

[4] The Royal Commission into Aged Care Quality and Safety. Final Report: Care, Dignity and Respect- List of Recommendations (https://agedcare.royalcommission.gov.au/sites/default/files/2021-03/final-report-recommendations.pdf). Canberra Commonwealth of Australia. 2021

[5] Ibrahim JE, Ranson DL, Bugeja L (2018). Premature deaths of nursing home residents: an epidemiological analysis. Med J Aust.

[6] Lloyd L, Banerjee A, Harrington C, Jacobsen F, Szebehely M (2014). It s a scandal! Comparing the causes and consequences of nursing home media scandals in five countries. Int J Social Soc Policy.

[7] Holroyd-Leduc JM, Laupacis A (2020). Continuing care and COVID-19: a Canadian tragedy that must not be allowed to happen again. Can Med Assoc J.

[8] McGilton KS, Escrig-Pinol A, Gordon A, Chu CH, Zuniga F, Sanchez MG, Boscart V, Meyer J, Corazzini KN, Jacinto AF, Spilsbury K, Backman A, Scales K, Fagertun A, Wu B, Edvardsson D, Lepore MJ, Leung AYM, Siegel EO, Noguchi-Watanabe M, Wang J, Bowers B (2020). Uncovering the Devaluation of Nursing Home Staff During COVID-19: Are We Fuelling the Next Health Care Crisis?. J Am Med Dir Assoc.

[9] Armstrong P (2018). Balancing the Tension in Long-Term Residential Care. Ageing Int.

[10] Gilbert AS, Garratt SM, Kosowicz L, Ostaszkiewicz J, Dow B (2021). Aged Care Residents' Perspectives on Quality of Care in Care Homes: A Systematic Review of Qualitative Evidence. Res Aging.

[11] Cleland J, Hutchinson C, Khadka J, Milte R, Ratcliffe J (2019). A Review of the Development and Application of Generic Preference-Based Instruments with the Older Population. Appl Health Econ Health Policy.

[12] Bulamu NB, Kaambwa B, Ratcliffe J (2015). A systematic review of instruments for measuring outcomes in economic evaluation within aged care. Health Qual Life Outcomes.

[13] Ratcliffe J, Cameron I, Lancsar E, Walker R, Milte R, Hutchinson CL, Swaffer K, Parker S (2019). Developing a new quality of life instrument with older people for economic evaluation in aged care: study protocol. BMJ Open.

[14] Khadka J, Ratcliffe J, Hutchinson C, Cleland J, Mulhern B, Lancsar E, Milte R. Assessing the construct validity of the Quality-of-Life-Aged Care Consumers (QOL-ACC): an aged care-specific quality-of-life measure. Qual Life Res. 2022;31(9):2849-65. 10.1007/s11136-022-03142-x PMC9181894 35680733

[15] Hutchinson C, Ratcliffe J, Cleland J, Walker R, Corlis M, Cornell V, Khadka J (2021). The integration of mixed methods data to develop the Quality of Life- Aged Care Consumers (QOL-ACC) measure. BMC Geriatr.

[16] Cleland J, Hutchinson C, McBain C, Walker R, Milte R, Khadka J, Ratcliffe J (2021). Developing dimensions for a new preference-based quality of life instrument for older people receiving aged care services in the community. Qual Life Res.

[17] Easton T, Milte R, Crotty M, Ratcliffe J (2017). Where's the evidence? a systematic review of economic analyses of residential aged care infrastructure. BMC Health Serv Res.

[18] Khadka J, Ratcliffe J, Chen G, Kumaran S, Milte R, Hutchinson C, Savvas S, Batchelor F (2020). A new measure of quality of care experience in aged care: psychometric assessment and validation of the Quality of Care Experience (QCE) questionnaire.

[19] Ratcliffe J, Bourke S, Li J, Mulhern B, Hutchinson C, Khadka J, Milte R, Lancsar E. Valuing the Quality of Life Aged Care Consumers (QOL-ACC) instrument for quality assessment and economic evaluation. Pharmacoeconomics. 2022:440(11):1069-79. 10.1007/s40273-022-01158-2 PMC9550725 35922616

[20] Malley JN, Towers AM, Netten AP, Brazier JE, Forder JE, Flynn T (2012). An assessment of the construct validity of the ASCOT measure of social care-related quality of life with older people. Health Qual Life Outcomes.

[21] Netten A, Burge P, Malley J, Potoglou D, Towers AM, Brazier J, Flynn T, Forder J, Wall B (2012). Outcomes of social care for adults: developing a preference-weighted measure. Health Technol Assess.

[22] Cleland J, Hutchinson C, Khadka J, Milte R, Ratcliffe J (2021). What defines quality of care for older people in aged care? A comprehensive literature review. Geriatr Gerontol Int.

[23] Gerlinger C, Bamber L, Leverkus F, Schwenke C, Haberland C, Schmidt G, Endrikat J (2019). Comparing the EQ-5D-5L utility index based on value sets of different countries: impact on the interpretation of clinical study results. BMC Res Notes.

[24] Norman R, Cronin P, Viney R (2013). A pilot discrete choice experiment to explore preferences for EQ-5D-5L health states. Appl Health Econ Health Policy.

[25] Mokkink LB, de Vet HCW, Prinsen CAC, Patrick DL, Alonso J, Bouter LM, Terwee CB (2018). COSMIN Risk of Bias checklist for systematic reviews of Patient-Reported Outcome Measures. Qual Life Res.

[26] Terwee CB, Prinsen CAC, Chiarotto A, Westerman MJ, Patrick DL, Alonso J, Bouter LM, de Vet HCW, Mokkink LB (2018). COSMIN methodology for evaluating the content validity of patient-reported outcome measures: a Delphi study. Qual Life Res.

[27] Lung T, Howard K, Etherton-Beer C, Sim M, Lewin G, Arendts G (2017). Comparison of the HUI3 and the EQ-5D-3L in a nursing home setting. PLoS ONE.

[28] Toh HJ, Yap P, Wee SL, Koh G, Luo N (2021). Feasibility and validity of EQ-5D-5L proxy by nurses in measuring health-related quality of life of nursing home residents. Qual Life Res.

[29] Abma IL, Rovers M, van der Wees PJ (2016). Appraising convergent validity of patient-reported outcome measures in systematic reviews: constructing hypotheses and interpreting outcomes. BMC Res Notes.

[30] Terwee CB, Bot SDM, de Boer MR, van der Windt DAWM, Knol DL, Dekker J, Bouter LA, de Vet HCW (2007). Quality criteria were proposed for measurement properties of health status questionnaires. J Clin Epidemiol.

[31] Gravesande J, Richardson J, Griffith L, Scott F (2019). Test-retest reliability, internal consistency, construct validity and factor structure of a falls risk perception questionnaire in older adults with type 2 diabetes mellitus: a prospective cohort study. Arch Physiother.

[32] Zijlmans EAO, Tijmstra J, van der Ark LA, Sijtsma K (2019). Item-Score Reliability as a Selection Tool in Test Construction. Front Psychol.

[33] Australian Bureau of Statistics. Socio-Economic Indexes of Areas (SEFIA) 2016 (https://www.abs.gov.au/websitedbs/censushome.nsf/home/seifa). Canberra. 2018

[34] Cleveland WS (1979). Robust locally weighted regression and smoothing scatterplots. J Am Stat Assco.

[35] Dinno A (2015). Nonparametric Pairwise Multiple Comparisons in Independent Groups using Dunn's Test. Stata J.

[36] Aspden T, Bradshaw SA, Playford ED, Riazi A (2014). Quality-of-life measures for use within care homes: a systematic review of their measurement properties. Age Ageing.

[37] Malley J, D'Amico F, Fernandez JL (2019). What is the relationship between the quality of care experience and quality of life outcomes? Some evidence from long-term home care in England. Soc Sci Med.

[38] Marten O, Brand L, Greiner W (2022). Feasibility of the EQ-5D in the elderly population: a systematic review of the literature. Qual Life Res.

[39] Rand S, Towers AM, Razik K, Turnpenny A, Bradshaw J, Caiels J, Smith N (2020). Feasibility, factor structure and construct validity of the easy-read Adult Social Care Outcomes Toolkit (ASCOT-ER)*. J Intellect Dev Disabil.

[40] Perez-Ros P, Martinez-Arnau FM (2020). EQ-5D-3L for Assessing Quality of Life in Older Nursing Home Residents with Cognitive Impairment. Life (Basel).

[41] Schwab CGG, Dichter MN, Berwig M (2018). Item distribution, internal consistency, and structural validity of the German version of the DEMQOL and DEMQOL-proxy. BMC Geriatr.

[42] Carrozzino D, Patierno C, Guidi J, Berrocal Montiel C, Cao J, Charlson ME, Christensen KS, Concato J, De Las Cuevas C, de Leon J, Eory A, Fleck MP, Furukawa TA, Horwitz RI, Nierenberg AA, Rafanelli C, Wang H, Wise TN, Sonino N, Fava GA (2021). Clinimetric Criteria for Patient-Reported Outcome Measures. Psychother Psychosom.

[43] Fleck MP, Carrozzino D, Fava GA (2019). The challenge of measurement in psychiatry: the lifetime accomplishments of Per Bech (1942–2018). Braz J Psychiatry.

