# Supplementary material for: Assessing feasibility, construct validity, and reliability of a new aged care-specific preference-based quality of life instrument: evidence from older Australians in residential aged care
Source: Health Qual Life Outcomes. 2022 Dec 1;20:159. doi: 10.1186/s12955-022-02065-y (PMC9713096; doi:10.1186/s12955-022-02065-y)
Supplement: Supplementary file 1 — Additional file 1: Table 1. Distribution of the Quality of Life- Aged Care Consumers (QOL-ACC) index scores by self-reported global health and quality of life items. Figure 1. Histograms showing distribution of scores of Quality of Life-Aged Care Consumers (QOL-ACC, 1A), Adult Social Care Outcome Tools (ASCOT, 1B), Quality of Care-Aged Care Consumers (QCE-ACC, 1C), EQ VAS (1D) and EQ-5D-5L (1E). [file 12955_2022_2065_MOESM1_ESM.docx]

**Additional file 1 , Table 1.** Distribution of the Quality of Life- Aged Care Consumers (QOL-ACC) index scores by self-reported global health and quality of life items.

| **Variables (N, %)** | **QOL-ACC scores** | | **Chi-squared, p value** |
| --- | --- | --- | --- |
|  | **Mean (SD)** | **Median (IQR)** |  |
| ***Self-reported health (200, 100%)*** |  |  | 49.4, p < 0.001 |
| Excellent (19, 9.5%) | 0.90 (0.10) | 0.92 (0.83-0.99) |  |
| Very good (48, 24.0%) | 0.85 (0.16) | 0.88 (0.78-0.95) |  |
| Good (66, 33.0%) | 0.76 (0.21) | 0.80 (0.67-0.90) |  |
| Fair (45, 22.5%) | 0.65 (0.24) | 0.71 (0.54-0.82) |  |
| Poor (22, 11.0%) | 0.50 (0.36) | 0.58 (0.28-0.79) |  |
| ***Self-reported quality of life (200,100%)*** |  |  | 64.4, p < 0.001 |
| Excellent (27, 13.5%) | 0.90 (0.11) | 0.95 (0.82-0.99) |  |
| Very good (52, 26.0%) | 0.82 (0.17) | 0.86 (0.77-0.95) |  |
| Good (70, 35.0%) | 0.77 (0.18) | 0.81 (0.68-0.89) |  |
| Fair (37, 18.5%) | 0.61 (0.24) | 0.68 (0.50-.78) |  |
| Poor (14, 7.0%) | 0.35 (0.37) | 0.40 (0.18-0.63) |  |

**Additional file 1, Figure 1:** Histograms showing distribution of scores of Quality of Life-Aged Care Consumers (QOL-ACC, 1A), Adult Social Care Outcome Tools (ASCOT, 1B), Quality of Care-Aged Care Consumers (QCE-ACC, 1C), EQ VAS (1D) and EQ-5D-5L (1E).

|  |  |
| --- | --- |
|  |  |
|  | |
